# Modified NEOMOD score as a neonatal mortality prediction tool in a medium‐income country: A validation diagnostic test study

**DOI:** 10.1002/hsr2.1065

**Published:** 2023-05-17

**Authors:** Laura Torres‐Canchala, Karen Molina, Mayra Barco, Laura Soto, Adriana Ballesteros, Alberto F. García

**Affiliations:** ^1^ Clinical Research Center Fundación Valle del Lili Cali Colombia; ^2^ Facultad de Ciencias de la Salud Universidad Icesi Cali Colombia; ^3^ Newborn Unit Fundación Valle del Lili Cali Colombia; ^4^ Department of Surgery Fundación Valle del Lili Cali Colombia

**Keywords:** infant, modified NEOMOD, mortality, neonatal intensive care, preterm, validation

## Abstract

**Background and Aims:**

Multiple organ dysfunction (MOD) is a potentially reversible physiological disorder that involves two or more systems. Modified NEOMOD (Neonatal Multiple Organ Dysfunction score) scale could be a useful instrument to measure MOD and predict mortality. Our aim was to validate modified NEOMOD in patients from a neonatal intensive care unit (NICU) of a middle‐income country.

**Methods:**

Diagnostic test study. Preterm newborns admitted NICU were included. Daily values were collected from birthday to Day 14. MOD was defined as at least one point in two or more systems. The lowest score is 0 and the maximum is 16. The outcome variable was mortality. Secondary outcomes were bronchopulmonary dysplasia, retinopathy of prematurity (ROP), late‐onset neonatal sepsis (LONS), intraventricular hemorrhage (IVH) and length of hospital stay. Area under the curve (AUC) and Hosmer–Lemeshow test were calculated to evaluate scale discrimination and calibration. Logistic regression was used to estimate the association between daily modified NEOMOD score and death.

**Results:**

We included 273 patients who met the inclusion criteria. MOD incidence was 74.4%. The median gestational age in patients with MOD was 30 (interquartile range [IQR]: 27–33) and in patients without MOD it was 32 (IQR: 31–33) (*p* < 0.001). There were 40 deaths (14.6%), 38 (18.7%) from the MOD group and 2 (2.9%) from non‐MOD group. On accumulated Day 7, AUC was 0.89 (95% confidence interval [CI]: 0.83–0.95). Modified NEOMOD had good calibration (*X* ^2^ = 2.94, *p* = 0.982). DBP (12.8% vs. 2.9%, *p* = 0.001), ROP (3.9% vs. 0%, *p* = 0.090), IVH (33% vs. 12.9%, *p* < 0.001), and LONS (36.5% vs. 8.6%, *p* < 0.001) frequency was higher in the MOD group than non‐MOD group. Length of hospital stay also was higher in MOD group (median 21 days [IQR 7–44] vs. median 5 days [IQR 4–9], *p* = 0.004).

**Conclusion:**

Modified NEOMOD scale presents good discrimination and calibration for death in preterm children. This scale could help in clinical decision‐making in real‐time.

## 1 BACKGROUND

Prematurity is defined as any birth occurring before 37 weeks of gestation. Worldwide, 15 million preterm babies are born each year, representing between 4% and 18% of all newborns.^1^ Prematurity is the main cause of death in children under 5 years of age, causing approximately one million deaths in 2015, of which three‐quarters have preventable causes. Therefore, prematurity is considered a public health problem.^2^, ^3^ Complications of prematurity are responsible for substantially higher morbidity and mortality rates in preterm infants than in term infants. The risk of complications is inversely proportional to the gestational age.^4^, ^5^

Multiple‐organ dysfunction (MOD) is defined as a potentially reversible physiological disorder that involves two or more systems that are not initially involved in the disorder responsible for admission to the neonatal intensive care unit (NICU).^6^ MOD increases the risk of morbidity and mortality in newborns, and its incidence is inversely proportional to gestational age.^7^ Although MOD is defined worldwide, problems persist when studying each patient individually, making it difficult for the medical team to make clinical decisions. Clinical decision‐making about newborns is complex due to its strong psychological and social components Clinical decision making for newborns is complex due to strong psychological and social components.^8^ Although patients' caregivers believe that decisions made about patients are in line with management guidelines, they believe that the will of the physician may also influence them.^9^ Factors such as (a) the difficulty of establishing a boundary between the management of patient complications and falling into medical futility, (b) the uncertainty about whether aggressive treatment will lead to a reasonably healthy child or a child with sequelae, and (c) the inability to determine what is an acceptable ratio between the probabilities of those two outcomes makes it difficult for the clinical team to make decisions and know what to offer and what not to offer to the patient's parents.^10^ The use of mortality prediction scores allows the team to have an objective tool based on the physiological state of the patient that will guide the direction of treatment.

Several scoring systems have been designed to perform this function in newborns. One of them, the Neonatal Multiple Organ Dysfunction (NEOMOD) score, was created by Janota et al.^11^ and modified by Çentikaya et al.^12^ This scoring system allows the analysis of eight organ systems, predicts mortality according to the level of involvement of each, is easy to use, and the required variables may be available in newborn units of high‐complexity hospitals in developing countries.^12^, ^13^ Unlike other existing mortality prediction scores,^14^, ^15^ where only variables related to birth and the first hours of life are considered, the NEOMOD score and its subsequent modification allow the probability of death to be estimated at any time during the first 28 days of life. Thus, clinically, it may come closer to the definition of MOD and provide accurate information to the treating clinician for timely and targeted decision‐making.

The modified NEOMOD scale was created with two objectives: the first is a real‐time objective diagnostic tool diagnosis of multiple organ dysfunction, and the second is a mortality prediction scale where the increase in the score of the scale implies a greater probability of death.^12^

Although the usefulness of NEOMOD has been extensively studied with good results, this scoring system should be evaluated in different population samples to confirm its universal applicability. The objective of this study is to evaluate the predictive capacity of the modified NEOMOD score for mortality in newborns with gestational age below 37 weeks in the NICU of a third‐level hospital in a developing country.

## 2 METHODS

Colombia is a middle‐income country with approximately 50 million inhabitants.^16^ Cali is the third largest city in Colombia, with approximately 2.6 million inhabitants, and is the capital of the department of Valle del Cauca. In 2017, the global birth rate of Cali was 12 per 1000 inhabitants, and the mortality rate was 9 per 1000 live births. Eleven percent of children who are born are diagnosed with low birth weight.^17^

Fundación Valle del Lili (FVL) is a third‐level hospital associated with the medical school of ICESI University. The Newborn Unit (NICU) has 41 NICU beds. An average of 2400 babies are born per year in FVL, of whom 46.5% are admitted to the NICU.

### 2.1 Study population and data collection

This is a diagnostic test validation study. All patients admitted to NICU from January 2018 to December 2019 were included. Children who were born at term (more than 36 weeks and 6 days of gestational age), stayed in the NICU for less than 24 h, were hospitalized in an intermediate‐care unit, had major malformations diagnosed by the clinical team, died within the first 24 h of life, or were born in another institution were excluded. Data were collected by retrospectively considering the patients who met the inclusion criteria from December 201 until reaching the needed sample size.

This was a diagnostic test validation study. All patients admitted to the NICU from January 2018 to December 2019 were included. Infants who were born at term (greater than 36 weeks and 6 days gestational age), stayed in the NICU less than 24 h, were hospitalized in an intermediate care unit, had major malformations diagnosed by the clinical team, died within the first 24 h of life, or were born at another institution were excluded. Patients who met the inclusion criteria were retrospectively recruited from December 2019 backward in time until the required sample size was reached.

### 2.2 Modified NEOMOD score

The NEOMOD scoring system was developed by Janota et al.^11^ and modified by Çetinkaya et al.^12^ The dysfunction of eight organ systems (central nervous system, cardiovascular system, kidneys, respiratory system, gastrointestinal system, hemocoagulation balance, acid–base balance, and microvascular system) is classified as moderate (1 point) or severe (2 points) every 24 h from Days 1 to 28 of life (Table 1). The maximum possible score of the modified NEOMOD is 16 points. The sensitivity of this scale is 84.1%, and the specificity is 78%, with an area under the receiver operating characteristic curve (AUC) of 0.943 ± 0.021.^12^

**Table 1:** Clinical characteristics and outcomes of newborns according to the presence of multiple‐organ dysfunction

Characteristics	Without multiple organ dysfunction (MOD)	With MOD	*p*‐value
	*n* = 70	*n* = 203	
Variables at birth			
Male sex, *n* (%)	39 (55.7)	115 (56.7)	0.892
Gestational age (weeks), median (interquartile range [IQR])	32 (31–33)	30 (27–33)	<0.001
Birth weight (g), median (IQR)	1664 (1497–1903)	1312 (906–1864)	<0.001
APGAR score, median (IQR)			
1 min	7 (6–8)	7 (5–8)	0.037
5 min	8 (8–9)	8 (7–9)	0.005
Maternal variables			
Maternal age (years), median (IQR)	24 (21–30)	25 (22–31)	0.689
Twins	16 (22.9)	39 (19.2)	0.512
Cesarean section, *n* (%)	44 (62.9)	144 (70.9)	0.208
Number of prenatal controls median (IQR)	5 (4–6)	4 (3–5)	0.028
Diseases of pregnancy, *n* (%)	50 (71.4)	153 (75.4)	0.308
Type of disease, *n* (%)			
Diabetes mellitus	2 (2.9)	6 (3)	0.731
Pre‐eclampsia	27 (38.6)	73 (36)	0.662
Placental abruption	3 (4.3)	8 (3.9)	0.702
Premature membrane rupture	14 (20)	39 (19.2)	0.886
Chorioamnionitis	17 (24.3)	34 (16.7)	0.163
Prolonged membrane rupture	1 (1.4)	16 (7.9)	0.129
Adolescent pregnancy	5 (7.1)	18 (8.9)	0.654
Complete steroid regimen, *n* (%)	28 (40)	96 (47.3)	0.367
Clinical outcomes			
Late neonatal sepsis, *n* (%)	6 (8.6)	74 (36.5)	<0.001
Intraventricular hemorrhage, *n* (%)	9 (12.9)	67 (33)	<0.001
IVH* grade, *n* (%)			
I	9 (100)	32 (47.8)	0.049
II	0 (0)	15 (22.4)	
III	0 (0)	12 (17.9)	
IV	0 (0)	8 (11.9)	
Retinopathy of prematurity (Grade III), *n* (%)	0 (0)	8 (3.9)	0.090
Persistent ductus arteriosus, *n* (%)	8 (11.4)	73 (36)	<0.001
Severe bronchopulmonary dysplasia, *n* (%)	2 (2.9)	26 (12.8)	0.001
Length of hospital stay (days), median (IQR)	11 (8–20)	21 (7–44)	0.004
Length of ICU stay (days), median (IQR)	5 (4–9)	12 (6–36)	<0.001
Death, *n* (%)	2 (2.9)	38 (18.7)	<0.001

The daily values required by the scoring system were collected from the day of birth until 14th day of life. The primary outcome variable was mortality. Secondary outcomes were bronchopulmonary dysplasia (BPD; defined as the need for supplemental oxygen for more than 28 days),^18^ retinopathy of prematurity (ROP) (stage 3 or higher),^19^ necrotizing enterocolitis (according to the Bell scale),^20^ intraventricular hemorrhage (IVH) (9), late onset sepsis and length of hospital stay.

### 2.3 Sample size

The sample size calculation was performed using the formula proposed by Riley et al. According to the validation study performed by Centinkaya et al.,^12^ the sensitivity of the scale is 84.1% and the specificity 78.0%. Based on this information, with a confidence level of 95% and a precision of 5%, with an eventual proportion of 74% and a minimum expected value of 68%, the required sample size is 264 patients.

### 2.4 Statistical analysis

The dichotomous variables are expressed as percentages. Continuous variables are expressed as median and interquartile range (IQR) or mean and standard deviation, depending on their distribution. The normality of the variables was estimated with the Shapiro‐Wilk test. The population was divided between newborns diagnosed and not diagnosed with MOD to compare the variables of interest. Fisher's exact test or the *χ* ^2^ test were used for categorical variables and the *t*‐test or Mann–Whitney test for continuous variables. The level of statistical significance adopted was *p* < 0.05.

The incidence of MOD was calculated as the number of cases of MOD detected divided by the total number of patients admitted to the NICU within the study period. Mortality was calculated in the groups with MOD and without MOD, taking as the numerator the number of deaths in each group and as the denominator the total population of each group.

The discrimination ability of the NEOMOD score was estimated by the area under the curve, with a confidence interval of 95%. The ROC curve was constructed taking the worst values recorded for each system per day over the entire patient follow‐up. Calibration was evaluated with hosmer‐lemeshow test. The analyses were performed with the statistical package Stata® 14.0 (Stata Corp). The study was approved by the nstitutional review board, given its retrospective nature, informed consent was exempted (# 1368).

## 3 RESULTS

**Figure 1:**
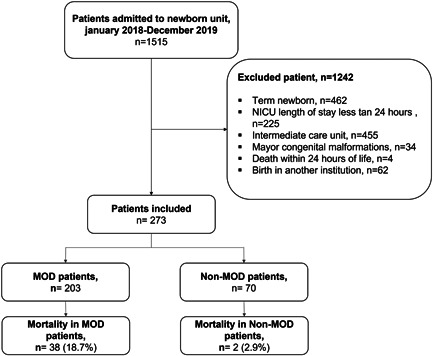
Selection patient flowchart

Figure 1 presents the flowchart of patient selection. Between January 2018 and December 2019, 1515 patients were admitted to the NICU. Of these, 1242 met the exclusion criteria. The study was thus conducted with 273 patients who met the criteria for inclusion in the study.

Table 1 describes the characteristics and clinical outcomes of patients with and without MOD. Mortality in the group without MOD was 1.9% and in the group with MOD was 38% (*p* < 0.001). Gestational age and birth weight were significantly lower in patients with MOD than their controls (*p* < 0.001 for both variables). The group with MOD presented significantly more cases of intraventricular hemorrhage (*p* = 0.001), ROP (*p* = 0.090), persistent ductus arteriosus (PDA) (*p* < 0.001), and severe BPD (*p* = 0.001). Patients with MOD often had moderate and severe Intraventricular hemorrhage, while only mild Intraventricular hemorrhage (*p* = 0.049) was present in the group without MOD.

The modified NEOMOD score was measured on 3169 days in the whole study sample. The median length of hospital stay for the survivors group was 18 days (IQR 9–38) and of nonsurvivors was 7 days (IQR 4–20) (*p* < 0.001).

**Figure 2:**
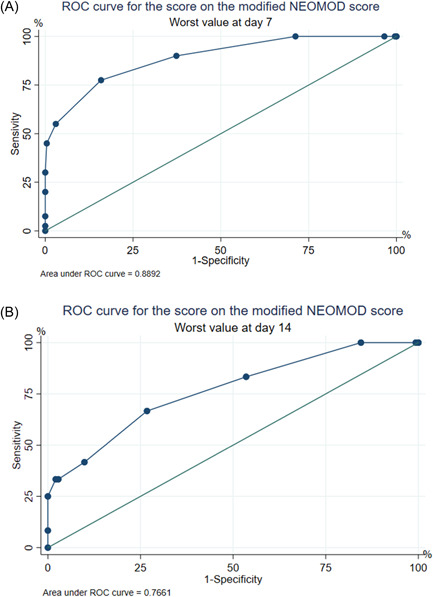
Receiver operating characteristic curve for the modified Neonatal Multiple Organ Dysfunction (NEOMOD) score; highest recorded score at Day 7 (A) and Day 14 (B)

Table 2 shows the univariate analysis of the daily modified NEOMOD score as a predictor of mortality. The scores of the modified NEOMOD score from Days 1 to 14 were significant prognostic factors for mortality. Given that MOD can gradually develop over days and does not necessarily appear on a single day, the predictive capacity of the score was evaluated by calculating it with the worst score of each variable during the first 7 days and until Day 14. The ROC curves of the cumulative scores at Day 7 and Day 14 are shown in Figure 2.

**Table 2:** Univariate analysis of the daily modified Neonatal Multiple Organ Dysfunction (NEOMOD) score as a predictor of mortality

Variable	Odd ratio	SE	95% confidence interval	*p*‐value
Day 1	2.4	0.56	1.5–3.79	<0.001
Day 2	1.8	0.34	1.26–2.6	0.001
Day 3	1.6	0.26	1.2–2.24	0.002
Day 4	1.7	0.26	1.23–2.25	0.001
Day 5	1.5	0.29	1.05–2.2	0.026
Day 6	1.6	0.28	1.16–2.28	0.005
Day 7	1.6	0.19	1.28–2.04	<0.001
Cumulative score at day 7	2.6	0.55	1.74–3.96	<0.001
Day 8	1.7	0.23	1.29–2.22	<0.001
Day 9	1.7	0.23	1.29–2.19	<0.001
Day 10	1.8	0.24	1.34–2.29	<0.001
Day 11	2.0	0.36	1.44–2.87	<0.001
Day 12	1.5	0.25	1.11–2.1	0.009
Day 13	1.8	0.28	1.32–2.43	<0.001
Day 14	2.1	0.34	1.48–2.84	<0.001
Cumulative score at day 14	2.1	0.40	1.46–3.07	<0.001

The median and IQR of the modified NEOMOD scores from Day 1 to Day 14 are presented in Table 3. The median cumulative score at Day 7 was 3 (IQR: 2–4) in survivors and 6 (IQR: 5–8) in nonsurvivors (*p* < 0.001). On Day 14, the median of the maximum cumulative score in survivors was 4 (IQR: 3–5), and in nonsurvivors it was 5 (IQR: 4–9) (*p* = 0.002).

**Table 3:** Daily score of the modified Neonatal Multiple Organ Dysfunction (NEOMOD) score: Discrimination and calibration

Day	Number of patients observed	NEOMOD score, survivors, median (IQR)	NEOMOD score, nonsurvivors, median (IQR)	*p*‐value	Discrimination: area under the curve (AUC) (95% confidence interval)	*χ* ^2^ calibration (*p*‐value)
1	273	2 (2–3)	4 (3–5)	<0.001	0.82 (0.77–0.88)	7.57 (0.182)
2	273	2 (1–3)	4 (3–6)	<0.001	0.81 (0.73–0.88)	9.85 (0.131)
3	273	2 (1–3)	4 (2–5)	<0.001	0.71 (0.6–0.82)	25.69 (0.002)
4	260	2 (1–3)	4 (3–6)	<0.001	0.82 (0.73–0.91)	8.78 (0.457)
5	244	0 (1–2)	3 (2–4)	<0.001	0.74 (0.63–0.85)	4.98 (0.663)
6	233	0 (1–2)	3 (2–4)	<0.001	0.79 (0.69–0.88)	6.85 (0.653)
7	219	0 (1‐–2)	1 (1–3)	0.005	0.74 (0.63–0.86)	5.53 (0.786)
Cumulative score at day 7	219	3 (2–4)	6 (5–8)	<0.001	0.89 (0.83–0.95)	2.94 (0.982)
8	212	0 (1–2)	3 (1–5)	<0.001	0.78 (0.68–0.90)	5.66 (0.685)
9	199	0 (1–2)	3 (1–5)	0.001	0.73 (0.60–0.87)	5.46 (0.605)
10	184	0 (1–2)	3 (1–5)	<0.001	0.77 (0.63–0.91)	6.10 (0.636)
11	178	0 (1–2)	3 (2–5)	<0.001	0.78 (0.64–0.93)	0.98 (0.964)
12	169	0 (1–2)	2 (2–3)	0.001	0.77 (0.64–0.90)	11.26 (0.08)
13	162	0 (1–2)	3 (2–5)	0.001	0.80 (0.67–0.94)	5.32 (0.62)
14	154	0 (1–2)	4 (3–8)	<0.001	0.84 (0.70–0.98)	9.16 (0.33)
Cumulative score at day 14	154	4 (3–5)	5 (4–9)	0.002	0.76 (0.61–0.91)	4.00 (0.86)

The AUC of modified NEOMOD score from Day 1 to Day 14 varied between 0.71 (95% CI: 0.60–0.82) and 0.84 (95% CI: 0.70–0.98), indicating moderate to good discrimination. The calibration assessed by the Hosmer‐Lemeshow test ranged between 25.69 (*p* = 0.002) and 0.98 (*p* = 0.964), indicating good calibration.

Table 4 presents the percentage probability of death according to the modified NEOMOD scale score. Univariate analysis data were used for the cumulative score at Day 7 (Appendix A1). Using the probability of death formula presented by Clark et al.^21^ and adapted by Letreurtre et al. in the pediatric ICU mortality scenario^22^ (Probability of death = 1/[1 + exp(‐logit[mortality]]), the probability of death according to each value of the modified NEOMOD scale was calculated.

**Table 4:** Probability of death according to the modified Neonatal Multiple Organ Dysfunction (NEOMOD) scale score

Score	Probability of death (%)
1	0
2	1
3	3
4	9
5	25
6	52
7	78
8	92
9	97
10	99
>11	100

## 4 DISCUSSION

The modified NEOMOD score had good discrimination and calibration in this study, which enrolled preterm newborns from a middle‐income country. The results confirm that the presence and severity of organ dysfunction can be evaluated with the modified NEOMOD score from Day 1 to Day 14. In addition, the modified NEOMOD score estimates death probability according to patient severity. Our findings show modified NEOMOD is an efficient tool to aid in making clinical decisions in real‐time. To our knowledge, this is the first study to validate the use of the modified NEOMOD score for preterm newborns in a middle‐income country.

This study not only shows that the modified NEOMOD score from Day 1 to Day 14 was a significant prognostic factor of death but also that mortality was significantly higher in children diagnosed with MOD, regardless of the severity.

Several mortality prediction scores have good prognostic performance and have been evaluated in different populations. Of these, the Clinical Risk Index for Babies (CRIB II) is the scoring system with the best risk adjustment in the NICU and presents an AUC of 0.84 in the first 30 days and the same value at 31–90  days.^14^, ^23^, ^24^, ^25^, ^26^ The revised version of the Score for Neonatal Acute Physiology (SNAP II) and its perinatal extension, SNAPPE II, present an AUC of 0.91 ± 0.01 to predict mortality.^15^ SNAPPE II has also shown good performance in predicting morbidities such as bronchopulmonary dysplasia and ROP.^27^ However, these scores have the major limitation that they can only be used with values collected at Day 1 of life. MOD can develop gradually over time,^6^ even more so in a newborn whose physiology is highly variable,^28^ for which reason it cannot be limited to independent daily evaluations.^7^

The sensitivity of the modified NEOMOD score was 84.1%, its specificity was 78%, and its AUC was 0.943 ± 0.021.^12^ This scale offers the advantage of measuring the probability through values obtained up to Day 30, thus eliminating the bias resulting from the high clinical variability observed in newborns during an ICU stay.

Although originally designed tools, such as the one created by Janota et al.^11^ and later modified by Çentikaya et al.,^12^ are developed with the necessary rigor to achieve external validity, multifactorial events can affect the tool's performance. In our study, the AUC of the modified NEOMOD score at Day 7 was 0.89 (95% confidence interval [CI]: 0.83–0.95) and at Day 14 was 0.76 (95% CI: 0.61–0.91). Although this finding suggests good performance of the tool, the Day‐7 AUC is five points lower than the best AUC estimated by Çetinkaya et al.^12^ This again reinforces the need to validate this type of instrument in the different populations that clinical teams serve. It is almost imperative that the validation of diagnostic and predictive tools be part of the quality processes of health services around the world. All this has one goal: to offer the best quality of care to patients and their families.

The modified NEOMOD score does not consider gestational age or birthweight as one of its variables. Mortality at 28 days is inversely proportional to gestational age, birthweight, and APGAR score at birth,^29^, ^30^, ^31^ which puts us at a potential disadvantage when trying to predict mortality. However, as stated by Çentikaya et al.^12^ and corroborated by our study, the modified NEOMOD score, by considering clinical variables measured daily or cumulatively (considering multiorgan failure as a process that develops over time), generates the necessary sensitivity to predict mortality, making it a feasible and rapid tool that can support clinical decision‐making in real time. The application of the modified NEOMOD score allowed us to dynamically evaluate the probability of death of preterm newborns admitted to the NICU, and it has the advantage of considering variables that are feasible to measure during daily routine care, which are key in the pathophysiology of MOD development.

When comparing patients with MOD vs without MOD, our study showed significant differences in the clinical outcomes of late neonatal sepsis, IVH, PDA, and BPD, which reflects the relevance of the nervous, cardiovascular, and respiratory systems.

### 4.1 Limitations and strengths

This study has several limitations. The first is the potential information bias due to its retrospective nature; after 14 days, fewer than half the data on the variables used in the score were available. Although the score allows replacing missing data with normal values, this can lead to an underestimation of the actual number of MODs. This study also has strengths. This is the first study to validate the modified NEOMOD score in the Latin American population of a middle‐income country, which allows it to be used in this population group in the NICUs of Latin American countries.

### 4.2 Next steps

In addition to the results and the certainty of being able to use the modified NEOMOD score in our population, the present study raises new questions and challenges for the scientific community. First, this study only evaluated the short‐term outcomes of patients. As proposed by Lee et al., it is necessary to conduct prospective studies to evaluate the performance of different tools for predicting mortality in the medium and long term.^25^ Second, at local level, it is important to compare the performance of the different scoring systems available in the literature to choose the best‐performing one in the given study population. Third, it is necessary to perform the validity studies in full‐term population.^6^ Fourth, it is necessary to evaluate the performance of this scale in patients referred from other hospitals. This study also reinforces the clinical relevance of validating the diagnostic tools used in the NICUs around the world.

## 5 CONCLUSION

The modified NEOMOD score is a feasible and efficient tool to predict mortality in preterm newborns in a middle‐income country. This study also reinforces the clinical relevance of validating the diagnostic tools used in the NICUs around the world.

## AUTHOR CONTRIBUTIONS

**Laura Torres‐Canchala**: Conceptualization; data curation; formal analysis; investigation; methodology; project administration; supervision; validation; visualization; writing – original draft; writing – review and editing. **Karen Molina**: Data curation; investigation; writing – original draft. **Mayra Barco**: Data curation; investigation; writing – original draft. **Laura Soto**: Data curation; investigation; writing – original draft. **Adriana Ballesteros**: Conceptualization; investigation; methodology; writing – original draft; writing – review and editing. **Alberto F. García**: Conceptualization; formal analysis; investigation; methodology; supervision; writing – original draft; writing – review and editing.

## CONFLICT OF INTEREST STATEMENT

The authors declare no conflict of interest.

## ETHICS STATEMENT

The study was approved by the institutional review board, and given its retrospective nature, informed consent was exempted (# 1368).

## TRANSPARENCY STATEMENT

The lead author Laura Torres‐Canchala affirms that this manuscript is an honest, accurate, and transparent account of the study being reported; that no important aspects of the study have been omitted; and that any discrepancies from the study as planned (and, if relevant, registered) have been explained.

## Data Availability

The datasets generated and/or analyzed during the current study are not publicly available to protect the rights of research participants according to Helsinki declaration but are available from the corresponding author on reasonable request.

## Appendix

 [Table hsr21065-tbl-0005]

**Table A1 hsr21065-tbl-0005:** Univariate logistic regression for death

Logistic regression					Number of obs	273
					LR *χ* ^2^(1)	96.74
					Prob > *χ* ^2^	0
Log‐likelihood = −65.367242					Pseudo *R* ^2^	0.4253

Death	Coefficient	Standard error	*z*	*p*‐value	IC 95%	
Cumulative Day 7 score	1.191976	0.1816664	6.56	0	0.8359164	1.548035
Cosntant.	−7.064417	0.8930124	−7.91	0	−8.814689	−5.314145

*Note*: These data were used to calculate probability of death according to modified Neonatal Multiple Organ Dysfunction (NEOMOD) score.
